# National Trends in Racial and Ethnic Disparities in Mortality from Mechanical Complications of Cardiac Valves and Grafts (1999–2020)

**DOI:** 10.3390/jcm14020562

**Published:** 2025-01-16

**Authors:** Ye In Christopher Kwon, David T. Zhu, Alan Lai, Andrew Min-Gi Park, Josue Chery, Zubair A. Hashmi

**Affiliations:** Division of Cardiothoracic Surgery, Department of Surgery, Pauley Heart Center, Virginia Commonwealth University School of Medicine, Richmond, VA 23298, USA

**Keywords:** racial disparities, ethnic disparities, coronary artery bypass and grafting, valvular heart disease, mechanical valves, CDC—center for disease control and prevention

## Abstract

**Background**: The volume of cardiac valve and coronary artery revascularization procedures is rising in the United States. This cross-sectional study explores ethnic disparities in mortality in cardiac surgery attributed to mechanical failures of implantable heart valves and coronary artery grafts. **Methods**: We used the CDC Wide-Ranging Online Data for Epidemiologic Research Multiple Causes of Death database to identify patients whose single cause of death was categorized by complications of cardiovascular prosthetic devices, implants, and grafts (ICD-10 code T82) between 1999 and 2020. The Joinpoint software (version 5.2.0, National Cancer Institute) was used to construct log-linear regression models to estimate the average annual percent changes in age-adjusted mortality (per 100,000). These patterns were compared and stratified by sex, age (0–44, 44–64, and 65 years or older), and US census regions between White, Black, Hispanic, non-Hispanic, American Indian, Alaskan Native, Asian American, and Pacific Islanders. **Results**: Age-adjusted mortality due to mechanical failures of cardiac implants and grafts declined across ethnicities from 2.21 (95% CI 2.16–2.27) in 1999 to 0.88 (95% CI 0.85–0.91) in 2020. Black populations (1.31 [95% CI 1.20–1.42]), both men (1.56 [95% CI 1.37–1.74]) and women (1.02 [95% CI 0.90–1.15]) experienced higher mortality in 2020 compared to all other ethnicities. This disparity was pronounced in younger groups (age 0–64), wherein age-adjusted mortality among Black populations (0.18 [95% CI 0.13–0.25]) more than doubled that of White populations (0.08 [95% CI 0.06–0.10]). **Conclusions**: Over the last two decades, age-adjusted mortality due to mechanical complications of cardiovascular implants has declined significantly. However, Black men and women, particularly younger patients, continue to experience higher death rates compared to other ethnicities.

## 1. Introduction

The volume of cardiac valve and revascularization procedures continues to rise in the United States, paralleling the growing burden of cardiovascular disease nationwide [1]. Coronary artery bypass grafting (CABG) is still the most common cardiac surgery performed today, and multiple-valve procedures are also being performed at increasing rates, with concomitant CABG and valve procedures becoming more popular in recent years [2,3]. While rare, prosthetic valve and graft dysfunction remains a serious complication in cardiac surgery. The most common causes of mechanical valve dysfunction are thought to be thrombosis and pannus formation, accounting for approximately 87.5% of case series [4]. This issue is not unique to mechanical valves, as thrombosis may also be responsible for an estimated 62.5% of bioprosthetic valve dysfunction, requiring reoperation [5]. In CABG, graft failure denotes complete obstruction, impeding the circulation of blood to the specific areas of the heart targeted for revascularization. Various factors, including morphological and functional characteristics of the graft, characteristics of the targeted vessel, the technique used for anastomosis or graft harvesting, and the patient’s atherosclerotic risks, may contribute to the intricate syndrome of graft failure [6]. However, the predominant mechanism of early graft failure, both in arterial and venous grafts, remains thrombosis followed by intimal hyperplasia and accelerated atherosclerosis [6]. This is likely further exacerbated by the patient’s co-existing systemic cardiovascular risk factors such as advanced age, hypertension, smoking, diabetes, and dyslipidemia [6,7]. Given the racial and ethnic disparities across these modifiable risk factors, along with inequalities in accessibility to interventions and treatments, ethnic minority groups continue to face particularly high cardiovascular disease burden and mortality [8,9]. In this setting, the impact of mechanical dysfunction after CABGs or valve replacements on these apparent disparities remains largely unknown. 

Racial disparities in cardiac surgical outcomes appear to be narrowing, yet patients from ethnic minority groups continue to face elevated mortality rates compared to their White counterparts [10,11]. Accordingly, there is a gap in studies examining long-term trends and social disparities in deaths from multifactorial complications of cardiothoracic surgery. This may ultimately hinder targeted clinical interventions aimed at improving outcomes for at-risk patient populations and mitigating structural racism within the care of underserved patients with heart diseases. Here, we aim to provide an overview of patterns in racial and ethnic differences in mortality due to mechanical dysfunction of implantable cardiac valves and coronary artery grafts.

## 2. Materials and Methods

### 2.1. Study Population

We analyzed the CDC Wide-Ranging Online Data for Epidemiologic Research Multiple Causes of Death (CDC WONDER) Multiple Causes of Death database for deaths associated with mechanical complications of cardiovascular prosthetic devices, implants, and grafts (International Classification of Diseases, Tenth Revision [ICD-10] diagnostic codes T-82) in the United States between 1990 and 2020. Crude and age-adjusted mortality rates (AAMRs) were calculated per 100,000 population and their 95% confidence intervals (CIs) were reported, examining trends in AAMRs between self-reported racial and ethnic groups (White, Black, Hispanic, non-Hispanic, American Indian, Alaskan Native [AIAN], Asian American and Pacific Islanders [AAPI]), sex (male vs. female), age (0–44, 45–64, 65 years or older), and United States census regions (Northeast, Midwest, South, and West). 

### 2.2. Statistical Analysis

Relative percent change (RPC) in AAMRs due to mechanical complications of cardiovascular prosthetic devices, implants, and grafts were calculated by comparing the calendar year 1990 to 2020. The 95% CI for RPC was calculated by sampling normal distributions based on the underlying AAMRs and their standard errors, defining the CI as the 2.5 and 97.5 percentiles of calculations based on those samples [12]. Log-linear regression models in Joinpoint software (version 5.2.0, National Cancer Institute, Bethesda, MD, USA) were used to estimate the average annual percent change (AAPC) for every ethnic/racial, sex, age, and US census group across these two time periods [13]. Briefly, by using mortality rates as input variables, this methodology delineates the year(s) in which a shift in trend occurs, computes the annual percent change (APC) rates between the identified trend-change intervals, and further assesses the AAPC during the whole study period. When there are no changes in trend (i.e., no join points), the APC remains constant and thus equals the AAPC. Conversely, the whole timeframe is partitioned according to the points where a shift in APCs is observed. Subsequently, AAPC is computed as a weighted mean of the estimated APC within each segment by using the lengths of the segments as weighting factors [13]. The quantity of joinpoints is determined by a permutation test via Monte Carlo resampling techniques, as described by Kim et al. [13]. Two-tailed statistical significance was set at *p* < 0.05. 

### 2.3. Ethical Statement

This study followed the Strengthening the Reporting of Observational Studies in Epidemiology (STROBE) guidelines. Due to the de-identified, publicly available nature of our data, this study was deemed exempt from the Virginia Commonwealth University Institutional Review Board.

## 3. Results

**Figure 1:**
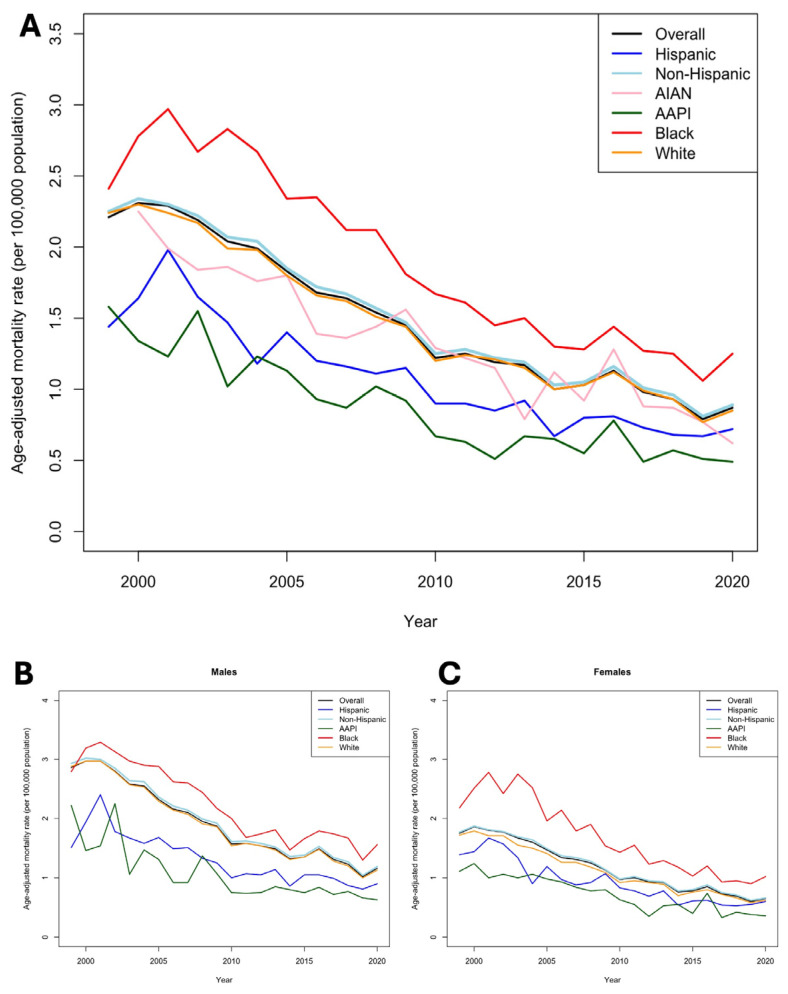
Trends in mortality due to mechanical failures of cardiac implants, valves, and grafts between 1999 and 2020. (**A**), Trends in overall age-adjusted mortality (per 100,000) between race/ethnicity. (**B**), Trends in age-adjusted mortality (per 100,000) between race/ethnicity stratified by male. (**C**), Trends in age-adjusted mortality (per 100,000) between race/ethnicity stratified by female.

Between 1999 and 2020, the AAMR per 100,000 population due to mechanical complications associated with implantable cardiac valves, arterial, and venous grafts for CABG declined, with an RPC of −60.18% (95% CI 51.39–61.23) across all ethnicities from 2.21 (95% CI 2.16–2.27) to 0.88 (95% CI 0.85–0.91) (Table 1; Figure 1A). Across all ethnicities, the rate of AAMR decline was −9.5% annually (*p* < 0.03). Black populations consistently experienced higher AAMRs across all years compared to other ethnicities (1.31 [95% CI 1.20–1.42]) and the lowest RPC of −46.53% (95% CI 46.21–46.62). However, the rate of AAMR decline among Black populations was the highest at −11.3% annually (*p* < 0.001). Notably, AAPI populations had the lowest AAMR in 2020 compared to all other ethnicities (0.48 [95% CI 0.39–0.57]) and experienced the highest decline in RPC of −69.62% (95% CI 69.05–70.00). However, the rate of AAMR decline among AAPI populations was the lowest at −6.2% yearly (*p* < 0.04). 

**Figure 2:**
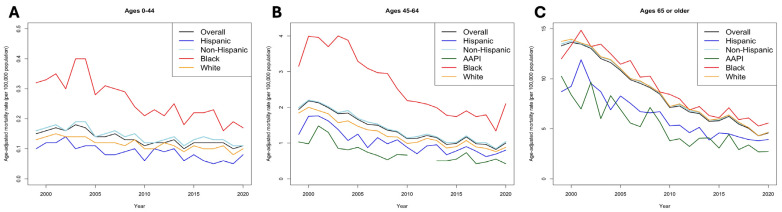
Trends in age-adjusted mortality (per 100,000) between race/ethnicity stratified by age groups. (**A**), Ages 0–44. (**B**), Ages 45–64. (**C**), Ages > 65 years.

Stratification by sex revealed key findings. Female patients saw a greater decline in AAMR (62.86% [95% CI 62.64–64.91]) compared to their male counterparts (−59.44% [95% CI 59.12–59.78]) across ethnicities (Figure 1B,C). Yet, in 2020, the male AAMR of 1.16 [95% CI 1.11–1.21] was nearly double that of females (0.65 [95% CI 0.61–0.68]). Sex-stratified AAMRs revealed that Black populations consistently have the highest AAMRs within both sexes (males: 1.56 [95% CI 1.37–1.74]; females: 1.02 [95% CI 0.90–1.15]) (Supplementary Table S1). These disparities were most pronounced in younger groups (ages 0–64), wherein the mortality rate among Black patients (0.18 [95% CI 0.13–0.25]) was more than two-fold higher than White patients (0.08 [95% CI 0.06–0.10]) (Figure 2A–C; Supplementary Table S2). 

Furthermore, age-stratified analyses revealed that elderly patients (>65 years) had a substantially higher mortality rate in 2020 (4.57 [95% CI 4.40–4.76]) compared to ages 45–64 (1.05 [95% CI 0.98–1.12]) and below 44 (0.1 [95% CI 0.09–0.12]) throughout all patient populations (Figure 2). However, populations aged >65 years also experienced the highest rate of decline during the study period (−65.59% [95% CI 65.23–65.94]) and the highest rate of change annually at −11.7% (*p* < 0.001). We did not find significant changes in AAPC among populations aged 0–44 years (−1.2%, *p* = 0.55). Among elderly patients, mortality rates appeared homogenous, although slightly lower among AAPI patients (2.7 [95% CI 2.11–3.40]) compared to other groups in 2020. 

**Figure 3:**
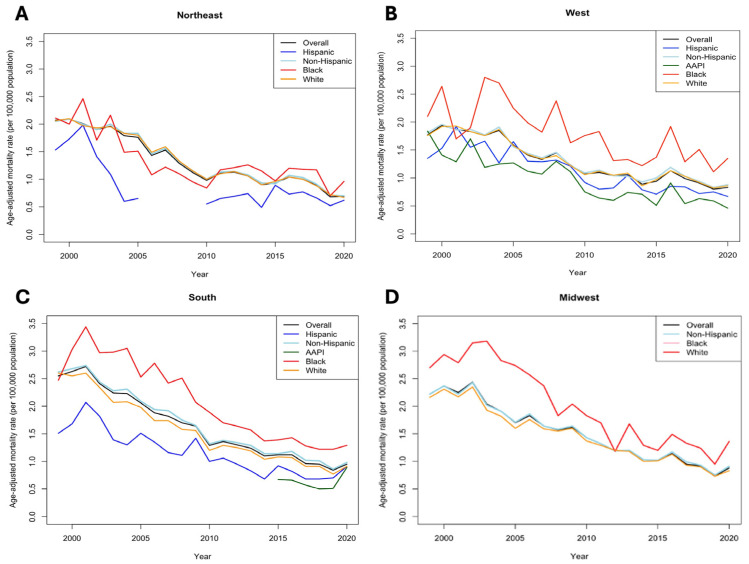
Trends in age-adjusted mortality (per 100,000) between race/ethnicity stratified by US census regions. (**A**), Northeast. (**B**), West. (**C**), South. (**D**), Midwest.

Finally, geographically stratified AAMRs revealed that patients across all ethnicities in Southern US census region, despite having the highest AAMR in 2020 at 0.95 (95% CI 0.9–1.0), experienced the largest changes in AAPC at −10.2% (*p* < 0.001). In contrast, we did not find significant changes in AAPC among patients in Midwestern US census regions (−2.1%, *p* = 0.53). Notably, Black populations in the Southern (1.29 [95% CI 1.15–1.44]), Northeastern (0.96 [95% CI 0.75–1.21]), and Midwestern (1.36 [95% CI 1.10–1.66]) US census regions consistently experienced the highest AAMRs in 2020 compared to other ethnicities (Figure 3). However, in the Western US census region, AAPI populations had the highest burden of AAMR (1.35 [95% CI 0.34–0.61]) in 2020 as well as the lowest RPC (−35.71% [95% CI 33.59–36.38]) between 1999 and 2020 compared to other ethnicities (Supplementary Table S3). 

## 4. Discussion

This comprehensive, national cross-sectional study examined mortality rates due to mechanical complications of cardiac valve replacements, bioprosthetic implants, and grafts across diverse racial and ethnic backgrounds. Further clinical and public health measures are urgently needed to address structural racism and social disparities experienced by Black populations that affect survival outcomes, complications, and recovery from cardiovascular surgeries. Our findings are largely consistent with the overarching patterns observed in cardiac surgery in the United States. Overall, AAMRs, due to mechanical complications of valve replacements and CABGs, have generally declined over the past two decades. Nevertheless, despite improvements in operative techniques and implementation of more equitable practices nationwide [14], Black men and women, especially those below 65 years of age, continue to experience disproportionately higher mortality. In contrast, AAPI populations experienced the lowest AAMRs, albeit with the smallest declines in mortality. This is likely attributed to the relatively lower burden of cardiac-related mortality among AAPI populations regardless of sex and age. 

Racial and ethnic disparities in the outcomes of cardiac surgery remain a controversial yet consistently debated and nuanced topic. A deeper understanding of these patterns is crucial to improve and optimize outcomes for a more diverse patient population undergoing cardiac revascularization procedures and valve replacements. The specific causes of these disparities remain difficult to identify due to the complex relationships between race or ethnicity and patient-level clinical characteristics. Variables exist across ethnicities regarding the burden and risk of developing coronary artery diseases, with some studies indicating that South Asian patients are at a higher risk of early and more severe complications from coronary artery diseases [15,16]. Other at-risk groups include certain ethnic populations from Eastern European [17], Middle Eastern [18], and North African [19] countries. These results may be significantly influenced by several other metabolic and cardiovascular comorbidities that occur at higher rates in these populations, including diabetes, hypertension, hyperlipidemia, and obesity [15,19]. Differences in primary dietary and exercise habits are also likely to play major roles in mediating the burden of cardiovascular disease or complications from cardiac surgery [20]. However, these factors remain difficult to control or account for in this dataset. Our study adds to the growing body of evidence that certain patient populations, even after receiving corrective surgeries for coronary artery or valvular diseases, remain at a higher risk of developing postoperative complications, particularly those related to mechanical thrombosis. These may not only be due to the disparities in baseline comorbidities or the burden of cardiovascular disease but also a factor of larger socioeconomic and hospital-level issues at hand.

In addition to inherent differences in patients’ clinical characteristics, disparities in outcomes may be a function of health system-level factors affecting the quality of care. Hospital quality, as measured by risk-adjusted mortality, has been shown to have a significant contribution to racial disparities in CABG outcomes, accounting for up to 66% of the differences in mortality rates between White and Black patients [21]. However, Black patients were more likely to undergo cardiac procedures at hospitals with higher risk-adjusted mortality [22], lower volumes [23], or surgeons with worse risk-standardized outcomes [24]. There are also other perioperative considerations that may contribute to the apparent racial disparities. Despite the high long-term patency of the internal mammary artery (IMA) grafts [25], the utilization of the IMA has been reported to be lower among Black patients [22]. The lack of access to adequate care may have other consequences for Black and minority patients, as late surgical referral has been associated with a higher likelihood of developing congestive heart failure and overall survival after mitral valve replacement [26]. In consideration of hospital-related factors, it may be warranted to expand evidence-based protocols like the Enhanced Recovery After Surgery (ERAS) program, which has demonstrated success in mitigating racial and socioeconomic disparities in aortic valve replacements [27]. ERAS programs have also been associated with reduced intensive care unit readmission and postoperative length of stay among Black and minority patients undergoing CABG [28]. Despite these reported benefits, it is possible that ERAS programs are not being implemented equally across hospitals, especially among hospitals with higher mortality statistics. 

Social and structural barriers may further exacerbate disparities in cardiac surgery outcomes and complications among Black patients and other racial and ethnic minorities. Socioeconomic status (SES) has long been considered a risk factor for mortality and adverse outcomes among CABG patients [14,29]. Patients from areas of high deprivation index (a granular measure of SES) are at a higher risk for in-hospital mortality after CABG [30]. Similarly, Medicaid insurance has been established as an independent risk factor of mortality compared to private insurance among patients undergoing CABG, in which Black and Hispanic patients under Medicaid insurance were more likely to be readmitted for complications after CABG [24,25]. While overall utilization of cardiac surgery after Medicaid expansion has increased, this has not been demonstrated among minority patients [31]. Furthermore, among minority patients, Medicaid expansion had no impact on mortality and complication rates, including postoperative arrhythmias [31]. These results signify that the relationship between increased access to care and improved outcomes is complex among racial minorities. The benefits of expanded coverage may be mitigated by several factors, including access to higher-volume, specialized hospitals [23] or improved referral pathways from primary care physicians to specialty surgeons [32]. However, addressing these potential inequities and the underlying causes of poorer outcomes may not be immediately resolved by merely expanding insurance coverage. Future studies must focus on better delineating the role of Medicaid expansion on the outcomes and specific complications of cardiac surgery, particularly among Black and minority patients. 

While quantifying differences in attitudes toward healthcare and care-seeking behavioral patterns between racial groups is difficult, prior studies have demonstrated that Black patients may experience longer delays in receiving timely valve replacements and CABGs [14,16]. Studies have also found that Black patients with symptomatic severe aortic stenosis were less likely to be treated with aortic valve replacement than their White counterparts [33]. This finding persists even after adjusting for age, biological sex, aortic calcification, aortic valve area, and stage of aortic stenosis, where Black patients with aortic valve disease were 54% less likely to be referred for valve replacement than White patients [34]. Furthermore, among patients who undergo transcatheter aortic valve replacements (TAVR), SES and racial disparities often intersected among Black patients, where for every $10,000 increase in income, the odds of undergoing transcatheter aortic valve replacement (TAVR) increased by 10% [35]. However, areas with higher minority populations often have fewer centers with high-volume TAVR programs [31,36]. These patterns underscore the myriads of challenges faced by Black and minority patients in accessing high-quality, affordable cardiac surgical care. Notably, these inequities have persisted even as aortic valve replacements have greatly expanded under the Medicare National Coverage Determination, which does not include racial equity as a target goal [37]. 

Inequitable access to postoperative cardiac rehabilitation may also contribute to the observed disparities. Referral to cardiac rehabilitation is currently supported by a Class 1 level of evidence, indicating enhanced long-term outcomes [38]. However, an analysis of a comprehensive national registry encompassing patients with coronary artery disease revealed that a significantly lower proportion of Black and Hispanic patients were referred for cardiac rehabilitation compared to non-Hispanic White patients [39]. Furthermore, it has been reported that the type of clinician significantly influences the disparities in cardiac rehabilitation referrals [40]. The same patient-related factors previously discussed, such as financial burdens, are likely to affect participation in cardiac rehabilitation. National registry studies have indicated diminished cardiac rehabilitation participation rates among underrepresented ethnic minority populations [41]. Notably, prior evidence demonstrates a positive association between income levels and participation in cardiac rehabilitation, whereby Black and Hispanic patients with the lowest household incomes exhibited the lowest rates of participation in cardiac rehabilitation [41]. Among Medicare fee-for-service beneficiaries eligible for cardiac rehabilitation in 2016, participation rates were notably lower for Hispanic and Black patients when juxtaposed with White patients (25.8%) [42].

Given the paramount importance of anticoagulation in preventing thrombosis and reducing the risk of mechanical complications, racial and ethnic disparities in access to anticoagulation, often mediated by SES factors, merit further discussion [43,44]. The Use of warfarin and, more recently, direct-acting oral anticoagulants (DOACs) has been shown to decrease the rate of thromboembolic events in patients who have undergone various cardiac surgeries or in patients with nonvalvular atrial fibrillation [45]. However, racial disparities in the use of DOACs persist in patients with underlying atrial fibrillation, with Black and Hispanic patients significantly less likely to receive DOACs compared to White patients [45]. A similar trend has been observed with warfarin administration, where Black patients in the Medicaid population are significantly less likely to fill warfarin prescriptions than White patients [46]. This may be further associated with SES factors such as lower household incomes among commercially insured Black and Hispanic populations [47,48]. Despite a steady increase in DOAC adoption across all ethnicities since 2012 [49], racial and SES inequity in access to DOAC may be contributing to increased rates of thrombosis, resulting in fatal complications of cardiac valves and grafts. Several reasons may contribute to this observed phenomenon. Within the framework of structural bias and underdiagnosis, Black patients may have limited awareness of their risk for thrombotic events or atrial fibrillation, likely reducing their chances of receiving guideline-based care [50]. The issue of patient-level trust, particularly in relation to new therapies, has been suggested as a potential factor influencing differential treatment; however, existing research does not substantiate this claim [51]. Additionally, the impact of unconscious bias among individual clinicians remains an insufficiently explored area that merits further research. A deeper understanding of the decision-making processes of physicians and other healthcare providers in prescribing DOACs could provide insight into the influence of race and ethnicity on anticoagulation treatments. To address these disparities, further clinical and public health interventions should expand guidelines for patients at a higher thrombotic risk undergoing mechanical valve replacements while increasing clinician familiarity with various anticoagulant therapies, awareness of fewer dietary restrictions, and reducing testing requirements among minority patients [48]. Additionally, efforts to improve hospital governance and strengthen the safety net hospital system across specific regions should be prioritized [52], accompanied by patient education on clinical risk factors and symptoms, as well as programs addressing socioeconomic and insurance barriers.

Moreover, the underlying causes of the persistently high AAMRs in the Southern US states, despite experiencing the largest decline in AAMRs during the study period, remain unclear. However, one pattern is clear: the Southern states perform the highest volume of major cardiac valve replacements and revascularization procedures. Prior studies have demonstrated that the Southern states perform the most CABGs, accounting for up to 48.5% as of 2018 [53]. Concurrently, the Southern states have also demonstrated the highest rates of reoperation after the index CABG, further suggesting that a variety of combinations in complications may be attributed to the observed AAMRs [54]. These trends are also similar among aortic valve replacements, where the South regions consistently perform a significantly higher proportion of aortic valve replacements in recent years [55]. In the modern era, it is unlikely that significant variations in surgeon expertise or practices across regions would play a major role in these patterns, especially given large efforts to standardize cardiac surgery training across the United States [56]. However, surgeon-to-surgeon variations are still seen as a major contributing factor in mortality and morbidity attributed to certain cardiac surgeries [57]. Furthermore, Southern states have historically had a larger proportion of Black and ethnic minority patients [58]. Thus, combined with the higher rates of poverty, lack of insurance, lower socioeconomic status, and older age in the South [58], the observed disparities among Black patients nationwide may be further amplified in the South.

Finally, despite lower cardiac surgery mortality rates among Asian American populations when analyzed as a single aggregated group, this approach obscures crucial disparities between specific disaggregated AAPI ethnic subgroups. For instance, prior evidence shows that Asian Indians and Filipino individuals experience a higher risk of cardiovascular conditions compared to other Asian Americans [59]. Thus, future research incorporating disaggregated AAPI groups is crucial to elucidate disparities and identify high-risk subgroups, aiming to inform culturally competent cardiac surgical care. Additionally, low health literacy among Asian Americans has also been shown to hinder risk factor prevention and timely diagnosis of underlying cardiovascular conditions [60]. Low health literacy has been associated with worse cardiovascular outcomes, including challenges with self-management of chronic diseases like hypertension and hyperlipidemia, which can accelerate the progression of atherosclerosis and valve degeneration, as well as challenges with following pre- and postoperative surgical guidelines [61]. Furthermore, low health literacy has been associated with insufficient knowledge of guidelines surrounding anticoagulant therapy, such as the discontinuing warfarin and DOACs, which may present an increased risk of intraoperative bleeding and postoperative thrombosis [62]. These may have important roles to play in mediating mechanical complications of cardiac surgery among AAPI subpopulations whose true burden of mortality may be blunted in our analysis. Hence, in addition to data disaggregation, further efforts are needed to address linguistic barriers, access to primary care, and expand access to culturally competent cardiac surgical care, among other factors that impede long-term recovery among AAPI cardiac surgery patients. 

### Limitations

Several limitations merit consideration when interpreting our findings. First, the cross-sectional design of this study limits the ability to establish causal relationships between the observed disparities and factors contributing to deaths due to mechanical complications of cardiac valve replacements and revascularization procedures. Similarly, we analyzed mortality from these complications as an underlying cause of death. While this method may give us more precise estimates of deaths directly linked to this complication, such reliance on death certificate data may come at the expense of potential misclassification or underreporting of deaths. Moreover, this provides limited specificity regarding the operative and procedural characteristics, including the types of valves and grafts. The objective of this study was to broadly overview and identify disparities in this complication of cardiac surgery. Thus, combined with the completely deidentified nature of the database, we did not link CDC-WONDER data with other clinical databases. Thus, we are unable to account for patient-level baseline clinical characteristics, including pre-existing comorbidities, prior cardiac surgery, and other perioperative risk factors that may confound outcomes. Thus, future studies should focus on identifying potential differences in clinical factors at index presentation across races, sex, and age in this population. Second, the ecological nature of the study may preclude further analyses of sociodemographic factors for individual patients, including income levels and insurance coverage. In a similar vein, our analyses cannot account for the potential geographic variations in reporting practices, preferences in surgical methods, and availabilities of certain newer valves or grafts. Third, the significant amount of data suppression on AIAN populations across ages and regions, as well as among AAPI populations in the Northeastern, Midwestern, and Southern US census regions, were also notable. Thus, our study findings should not be generalized to the AIAN populations at large and warrant more focused data collection in these minority groups. Finally, the reliance on the ICD-10 code T82 lends our analysis to significant limitations. Since ICD-10 codes were designed for billing purposes, issues related to the validity of these codes, including potential variabilities in coding practices or misclassification in the causes of death at the level of the individual surgeon or the larger health system, may not reflect the true clinical outcomes. Ultimately, these racial and ethnic disparities underscore the urgent need for targeted clinical guidelines and policies to reduce preventable mortality in at-risk patients undergoing high-risk cardiac surgery. Further research to identify the structural barriers, such as affordability and accessibility to specialized facilities, is imperative for equitable cardiac surgical outcomes among medically underserved populations. Additionally, future studies should disaggregate Asian American subgroups, given that certain populations, such as South Asians, have a disproportionately large burden of cardiovascular conditions and could benefit from expanded public health efforts to address structural barriers.

## 5. Conclusions

Overall, age-adjusted mortality rates due to mechanical complications of cardiac valves, implants, and grafts have continued to decline from 1999 to 2020. However, Black men and women, particularly patients under 65 years, continue to experience disproportionately higher mortality after coronary artery bypass grafting and valvular procedures. These findings highlight potential structural disparities in valvular and cardiac revascularization procedures. To close the mortality gap following cardiac surgery among Black individuals, addressing both clinical and structural barriers is essential. This includes refining preoperative risk stratification models to account for social determinants of health, such as inequitable access to care and income disparities, which disproportionately affect Black patients. Moreover, reducing postoperative mortality requires targeted interventions aimed at improving perioperative management, increasing access to rehabilitation services, and expanding guidelines for culturally competent cardiac surgery care, which could mitigate adverse outcomes and narrow racial and ethnic disparities in cardiac surgery-related mortality.

## Figures and Tables

**Table 1:** Age-adjusted mortality rates attributable to mechanical complications of cardiovascular prosthetic devices, implants, and grafts in the United States, 1999–2020.

	Age-Adjusted Mortality Rate per 100,000 (95% CI)		Relative Percent Change (95% CI)	Annual Average Percent Change (*p*-Value)
	1999	2020		
Total	2.21 (2.16–2.27)	0.88 (1.05–0.88)	−60.18 (51.39–61.23)	−9.5 (*p* = 0.013)
Race and Ethnic Group				
White	2.26 (2.2–2.32)	0.83 (0.8–0.86)	−63.27 (62.93–63.63)	−9.7 (*p* < 0.001)
Black	2.45 (2.24–2.64)	1.31 (1.2–1.42)	−46.53 (46.21–46.62)	−11.3 (*p* < 0.001)
Hispanic	1.44 (1.24–1.64)	0.72 (0.63–0.8)	−50.00 (49.19–51.22)	−6.8 (*p* = 0.027)
Non-Hispanic	2.26 (2.2–2.32)	0.89 (0.85–0.92)	−60.62 (60.34–61.36)	−9.3 (*p* < 0.001)
AIAN	2.37 (1.61–3.36)	0.95 (0.61–1.42)	−59.92 (57.74–62.11)	−9.2 (*p* < 0.001)
AAPI	1.58 (1.26–1.9)	0.48 (0.39–0.57)	−69.62 (69.05–70.00)	−6.2 (*p* = 0.036)
Sex				
Female	1.75 (1.69–1.82)	0.65 (0.61–0.68)	−62.86 (62.64–63.91)	−6.2 (*p* = 0.031)
Male	2.86 (2.76–2.96)	1.16 (1.11–1.21)	−59.44 (59.12–59.78)	−8.9 (*p* = 0.0056)
Age Group (Years)				
0–44	0.15 (0.13–0.17)	0.1 (0.09–0.12)	−33.33 (29.41–33.76)	−1.2 (*p* = 0.55)
45–64	1.96 (1.85–2.08)	1.05 (0.98–1.12)	−46.43 (46.15–47.03)	−3.5 (*p* = 0.032)
>65	13.31 (12.92–13.69)	4.58 (4.4–4.76)	−65.59 (65.23–65.94)	−11.7 (*p* < 0.001)
US Census Regions				
Northeast	2.07 (1.95–2.18)	0.69 (0.63–0.76)	−66.67 (65.14–67.69)	−8.9 (*p* < 0.001)
Midwest	2.22 (2.11–2.34)	0.88 (0.82–0.94)	−60.36 (59.83–61.14)	−2.1 (*p* = 0.53)
South	2.55 (2.45–2.65)	0.95 (0.9–1.0)	−62.75 (62.26–63.27)	−10.2 (*p* < 0.001)
West	1.77 (1.66–1.88)	0.83 (0.77–0.89)	−53.11 (52.66–53.61)	−6.3 (*p* = 0.033)

## Data Availability

The original data presented in the study are openly available in the CDC Wide-Ranging Online Data for Epidemiologic Research Multiple Causes of Death (CDC WONDER) Multiple Causes of Death database.

## Supplementary Materials

The following supporting information can be downloaded at https://www.mdpi.com/article/10.3390/jcm14020562/s1, Table S1: Age-adjusted mortality rates stratified by sex and ethnicity, 1999–2020. Table S2: Age-adjusted mortality rates stratified by age and ethnicity, 1999–2020. Table S3: Age-adjusted mortality rates stratified by the US census regions and ethnicity, 1999–2020.
