# Supplementary material for: National Trends in Racial and Ethnic Disparities in Mortality from Mechanical Complications of Cardiac Valves and Grafts (1999–2020)
Source: J Clin Med. 2025 Jan 16;14(2):562. doi: 10.3390/jcm14020562 (PMC11765941; doi:10.3390/jcm14020562)
Supplement: Supplementary file 1 [file jcm-14-00562-s001.zip › jcm-3350823-supplementary.pdf]

Supplementary Tables

Table S1. Age-adjusted mortality rates stratified by sex and ethnicity, 1999-2020.

	**FEMALES**			**MALES**		
	Age-adjusted mortality rate per 100,000 (95% CI)		Relative percent change (95% CI)	Age-adjusted mortality rate per 100,000 (95% CI)		Relative percent change (95% CI)
	**1999**	**2020**		**1999**	**2020**	
**RACE AND ETHNIC GROUP**						
**WHITE**	1.7 (1.63-1.77)	0.62 (0.58-0.66)	-63.52 (62.71-64.41)	2.89 (2.78-2.99)	1.11 (1.06-1.17)	-61.59 (60.86-61.87)
**BLACK**	2.18 (1.94-2.42)	1.04 (0.9-1.15)	-53.21(52.47-53.61)	2.8 (2.46-3.14)	1.56 (1.37-1.74)	-44.28 (44.20-44.58)
**HISPANIC**	1.39 (1.12-1.65)	0.58 (0.49-0.68)	-58.27 (56.25-58.78)	1.5 (1.2-1.86)	0.89 (0.75-1.04)	-40.67 (37.50-44.08)
**NON-HISPANIC**	1.76 (1.69-1.83)	0.67 (0.63-0.71)	-61.93 (61.20-62.72)	2.92 (2.82-3.02)	1.18 (1.13-1.24)	-59.58 (58.94-58.92)
**AIAN**	1.83 (1.1-2.86)	1.02 (0.63-1.6)	-43.16 (42.72-44.05)	2.52 (1.49-3.98)	1.08 (0.66-1.67)	-57.14 (55.70-58.04)
**AAPI**	1.11 (0.79-1.51)	0.37 (0.27-0.5)	-66.67 (65.82-66.88)	2.21 (1.67-2.88)	0.62 (0.48-0.8)	-71.94 (71.25-72.22)

Table S2. Age-adjusted mortality rates stratified by age and ethnicity, 1999-2020.

	**0-44 YEARS**			**45-64 YEARS**			**>65 YEARS**		
	Age-adjusted mortality rate per 100,000 (95% CI)		Relative percent change (95% CI)	Age-adjusted mortality rate per 100,000 (95% CI)		Relative percent change (95% CI)	Age-adjusted mortality rate per 100,000 (95% CI)		Relative percent change (95% CI)
	**1999**	**2020**		**1999**	**2020**		**1999**	**2020**	
**RACE AND ETHNIC GROUP**									
**WHITE**	0.15 (0.13-0.17)	0.08 (0.06-0.10)	-46.67 (46.66-53.84)	1.86 (1.75-1.98)	0.85 (0.79-0.92)	-54.30 (53.53-54.86)	13.57 (13.16-13.98)	4.56 (4.36-4.76)	-66.39 (65.95-66.87)
**BLACK**	0.34 (0.26-0.42)	0.18 (0.13-0.25)	-47.05 (47.05-50.00)	3.14 (2.7-3.58)	2.09 (1.82-2.36)	-33.43 (32.59-34.07)	11.89 (10.61-13.17)	5.34 (4.69-5.98)	-55.08 (54.59-55.79)
**HISPANIC**	0.1 (0.06-0.16)	0.07 (0.05-0.10)	-30.00 (16.67-31.02)	1.21 (0.91-1.58)	0.78 (0.62-0.93)	-35.53 (31.87-41.14)	8.72 (7.23-10.2)	3.94 (3.35-4.54)	-54.81 (53.66-55.59)
**NON-HISPANIC**	0.17 (0.15-0.20)	0.1 (0.09-0.12)	-41.17 (40.00-41.17)	2.00 (1.89-2.12)	1.05 (0.98-1.13)	-47.50 (46.69-48.15)	13.49 (13.09-13.88)	4.62 (4.43-4.81)	-65.75 (65.35-66.16)
**AIAN**	-	-	-	-	-	-	-	-	-
**AAPI**	-	-	-	1.05 (0.66-1.59)	0.40 (0.25-0.59)	-61.90 (61.12-62.89)	10.27 (8.05-12.91)	2.70 (2.11-3.40)	-73.71 (73.66-73.78)

Table S3. Age-adjusted mortality rates stratified by the US census regions and ethnicity, 1999-2020.

	**NORTHEAST**			**MIDWEST**			**SOUTH**			**WEST**		
	Age-adjusted mortality rate per 100,000 (95% CI)		Relative percent change (95% CI)	Age-adjusted mortality rate per 100,000 (95% CI)		Relative percent change (95% CI)	Age-adjusted mortality rate per 100,000 (95% CI)		Relative percent change (95% CI)	Age-adjusted mortality rate per 100,000 (95% CI)		Relative percent change (95% CI)
	**1999**	**2020**		**1999**	**2020**		**1999**	**2020**		**1999**	**2020**	
**RACE AND ETHNIC GROUP**												
**WHITE**	2.06 (1.94-2.19)	0.67 (0.61-0.75)	-67.47 (66.21-68.55)	2.16 (2.04-2.27)	0.83 (1.77-0.90)	-61.57 (60.35-62.25)	2.60 (2.49-2.71)	0.91 (0.86-0.97)	-65.00 (64.20-65.46)	1.76 (1.64-1.52)	0.86 (0.80-0.93)	-51.13 (50.26-51.21)
**BLACK**	2.11 (1.69-2.53)	0.96 (0.75-1.21)	-54.50 (52.17-55.62)	2.70 (2.22-3.18)	1.36 (1.10-1.66)	-49.62 (47.79-50.45)	2.47 (2.20-2.74)	1.29 (1.15-1.44)	-47.77 (47.44-47.44)	1.84 (1.52-2.83)	0.46 (0.01-0.77)	-75.00 (72.79-99.34)
**HISPANIC**	1.53 (1.04-2.17)	0.62 (0.44-0.85)	-59.47 (57.69-60.83)	-	-	-	1.51 (1.20-1.88)	0.91 (0.76-1.06)	-39.73 (36.67-43.62)	1.35 (1.06-1.71)	0.67 (0.54-0.80)	-50.37 (49.05-53.21)
**NON-HISPANIC**	2.05 (1.93-2.17)	0.70 (0.64-0.77)	-65.85 (64.52-66.84)	2.22 (2.11-2.34)	0.91 (0.84-0.97)	-59.01 (58.55-60.19)	2.62 (2.51-2.72)	0.98 (0.92-1.03)	-62.59 (62.13-63.35)	1.81 (1.69-1.93)	0.88 (0.81-0.95)	-51.38 (50.77-52.07)
**AIAN**	-	-	-	-	-	-	-	-	-	2.71 (1.61-4.29)	1.26 (0.45-1.45)	-53.51 (52.04-56.20)
**AAPI**	-	-	-	-	-	-	-	-	-	2.10 (1.44-2.31)	1.35 (0.34-0.61)	-35.71 (33.59-36.38)
